# Is alcohol dependence best viewed as a chronic relapsing disorder?

**DOI:** 10.1111/j.1360-0443.2011.03583.x

**Published:** 2012-01

**Authors:** John A Cunningham, Jim McCambridge

**Affiliations:** 1 Centre for Addiction and Mental HealthToronto, Ontario, Canada; 2 University of TorontoToronto, Ontario, Canada; 3 Faculty of Public Health and Policy, London School of Hygiene and Tropical MedicineLondon, UK

**Keywords:** Alcohol dependence, alcoholism, biomedicalization, brain disease, chronic relapsing disorder, DSM-V, epidemiology, treatment

## Abstract

This ‘For Debate’ paper starts by recognizing the growing trend towards considering alcohol dependence as a chronic relapsing disorder. We argue that the adoption of this model results from focusing on those in treatment for alcohol dependence rather than considering the larger number of people in the general population who meet criteria for alcohol dependence at some point in their lives. The majority of the general population who ever experience alcohol dependence do not behave as though they have a chronic relapsing disorder: they do not seek treatment, resolve their dependence themselves and do not relapse repeatedly. We suggest that caution is therefore needed in using the chronic relapsing disorder label. Our primary concerns are that this formulation privileges biological aspects of dependence to the detriment of psychological and social contributions, it inhibits much-needed developments in understanding alcohol dependence and leads to inefficient distributions of public health and clinical care resources for alcohol dependence. We invite debate on this issue.

## INTRODUCTION

There appears to be a growing acceptance that alcohol dependence is a chronic relapsing disorder [1–8]; but is this description an accurate label to apply? Does it capture the core features of this problem [9–12], and is it useful? In this ‘For Debate’ paper we will explore why this term may be attractive, as well as its limitations. We suggest that the majority of people with alcohol dependence do not behave as though they have a chronic relapsing disorder. Further, we will argue that conceiving of alcohol dependence as a chronic relapsing disorder may be detrimental to our attempts to understand the nature of this phenomenon, and to decisions on what to do about it.

## WHERE DOES THE CONCEPT COME FROM?

The principal source of the chronic relapsing disorder model of alcohol dependence may be neurobiology research. There have been several high-profile papers, published by leaders in the field in influential journals [1–4], describing addiction as a brain disease and as a chronic relapsing disorder, and the two can be easily conflated. Neurobiology has made great strides in understanding the impact of substances of abuse on the brain, and these findings have advanced a fundamentally biological explanation of addiction. However, as has been discussed eloquently by Kalant [13], there are limitations to how far neurobiology can take us towards understanding a problem that has social and psychological as well as biological roots. The neurobiological chronic relapsing disorder perspective tends towards reductionist rather than integrative conceptions of dependence. In addition, we argue that this emphasis on the biological component of an intrinsically interdisciplinary problem results from and reinforces an emphasis on the more severe end of the continuum of dependence problems. It is usually those with severe dependence who are under study in neuroscience. This focus may narrow rather than broaden our understanding, as it implies a categorical separation of the addicted (or the alcoholic) from the non-addicted (the favoured terms within neuroscience), because some people have this problem while others do not. This is at odds with clinically based attention to severity of dependence, prevailing conceptions of the nature of dependence itself, and also what is known in population health sciences.

Other strands of research have also contributed to the adoption of the conception of alcohol dependence as a chronic relapsing disorder. McLellan [14,15] makes the valid point that people with alcohol and drug dependence who show up in specialized addictions settings will often relapse repeatedly. An analogy is drawn with other health conditions, such as hypertension, which is regarded in a completely different fashion by society at large, and by the medical community, yet displays a similar chronicity to that seen in some people with alcohol dependence. Further, the success of treatment for hypertension appears to be judged by a different set of criteria than those applied to alcohol treatment. The latter is usually judged as successful if a single treatment course leads to long-term or permanent remission and the former is judged successful if the hypertensive patient has reduced symptoms during the time they are taking the treatment. McLellan is careful, however, to specifically avoid a discussion of the nature of dependence or addiction in an editorial in this journal [15] and to concentrate instead on the ways in which treatment responses are conceptualized and evaluated. The *JAMA* paper [14] is additionally concerned with advocacy that dependence is suitable for insurance, just like other chronic medical problems, and this paper becomes cited as providing evidential support to the chronic relapsing disorder formulation (e.g. [2]). McLellan's work also clearly embraces the need to provide a much broader continuum of care for those with addictions concerns [16,17].

## AN ALTERNATE VIEW

Our approach to alcohol dependence comes primarily from a population health perspective. That is, when the course of alcohol dependence is examined for the entire population of people who meet criteria for this disorder, do they behave as though they have a chronic relapsing disorder? The majority of people who meet criteria for alcohol dependence at some point in their life: (i) do not seek treatment [18–21]; (ii) resolve their alcohol dependence without any formal treatment or similar help [19,22,23]; and (iii) do not relapse repeatedly to alcohol dependence [19,22,24,25]. We stress here that this is not to say that some people with alcohol dependence do not relapse repeatedly and that a chronic care model of treatment would be ill-advised for this subpopulation—just that most people who experience alcohol dependence do not relapse again and again. If we want to understand the nature of alcohol dependence, or other addictive behaviours, then it is important to examine the full range of people experiencing the problem, thus including but not restricted to those with severe dependence only.

## COMMON GROUND

Is there a middle ground between these two views of alcohol dependence that may be appreciated when one considers the component terms—chronic, relapsing and disorder? For many people who experience alcohol dependence, there is indeed chronicity in the sense that this is not just a short-lived episode responsive to some sort of quick fix. Alcohol dependence usually takes many years to develop and be maintained [26–34] before it is subsequently overcome by most people on their own [19,22,23]. Drinking behaviour is dynamic and modified by a multitude of influences at different levels of intervention among those who are alcohol-dependent. Difficulties in controlling consumption are characteristic of many of those who have dependence and are fundamental to our understanding of it, and great suffering is involved in their personal struggles to limit the associated harms. Relapse in the sense of setbacks in these struggles is not at all unusual. Finally, alcohol dependence is clearly a disorder in diagnostic terms and disordered in the sense that it conflicts with deeply held values and goals and does damage to them [35].

It is not difficult to see why viewing alcohol dependence as a chronic relapsing disorder has appeal, as the formulation attests to important aspects of the phenomenon. Therein lies its limitation, as it does not capture accurately the apparent experience of most people affected by alcohol dependence, and thus potentially obscures rather than illuminates the full range of problems of dependence. It is almost as though the term ‘chronic relapsing disorder’ takes on a life of its own, holding more meaning than when its constituent terms are used in isolation. When applied by leaders of the field in non-specialist addiction journals, it is used to communicate the essential nature of dependence [1,2]. Finer-grained attention to the long-term course of the behaviour in the general population as observed in epidemiological studies provides a dramatically different and more heterogeneous picture from that attained in biologically orientated clinical research studies in treatment populations. This disjoint between the understanding of what is alcohol dependence in clinical and general population settings is at the root of our concern with adopting the model of a chronic relapsing disorder for alcohol dependence.

## THIS TENSION IS NOT NEW

The different perspectives held by addictions researchers with a clinical versus a population health perspective are not new. Room [36] referred to these as the two worlds of alcohol problems at a time early in the development of the modern epidemiological study of alcohol dependence. These different perspectives have far-reaching consequences, because the picture that is derived of alcohol dependence is very different depending on the researcher's or policy maker's orientation. Fundamentally, it comes down to how we define the population with alcohol dependence—does it consist of only those who are seen in treatment or does it comprise everyone who meets criteria for alcohol dependence in the general population? If one restricts the perspective to just those in specialized addictions services then it may be useful to regard alcohol dependence as a chronic relapsing disorder, although even here severity of dependence has long been accepted to be a key determinant of likelihood of relapse [37]. When the scope of alcohol dependence is widened to include all those who meet diagnostic criteria within an epidemiological survey then the concept of a chronic relapsing disorder does not seem as applicable. It is important to be clear that this broader epidemiological perspective emphasizes the need to be appreciative of the full continuum of alcohol problems (up to and including severe alcohol dependence), and not any sort of false dichotomy between those who alcohol dependent and those who are not.

## WHY DOES THIS MATTER?

### For public health

The danger of conceptualizing alcohol dependence as a chronic relapsing disorder is that it directs attention to a subgroup of those with severe dependence, and thus potentially undermines public health strategic responses for both those with dependence and those whose drinking is harmful or hazardous rather than dependent [38]. A consideration of the entire continuum of alcohol problems (including both those at risk of problems and those with alcohol dependence) is the necessary context for a population perspective on alcohol control, not least because population-level interventions are an important source of benefit for dependent drinkers as well as for other at-risk drinkers [39,40]. Further, it is this larger population of at-risk drinkers who cause the majority of costs to society that result from alcohol consumption, simply because of their numbers (referred to as the prevention paradox; [41–43]). The best evidence regarding ways to reduce the prevalence of alcohol problems in our society rests almost entirely with public health initiatives (such as reducing availability, taxation, drinking and driving legislation and the provision of brief interventions; [40]). Any model of alcohol dependence that takes us away from this recognition of the primary importance of public health interventions in the prevention and management of alcohol problems in the general population may thus impair our ability to effectively address these significant societal concerns.

### For clinical care

There are a number of reasons why it matters whether or not we adopt a chronic relapsing disorder model of alcohol dependence for clinical care. A concentration on the chronic relapsing nature of alcohol dependence may lead to a preponderance of resources going towards those with severe dependence and other co-occurring health (including mental health) issues. The appropriate balance of resources requires an appreciation that dependence occurs on a continuum in which there are many more people with mild to moderate than severe behavioural dysfunction [38,44]. This continuum of severity does not in any way imply a necessary progression from early and mild to late and severe. A range of services of varying intensities is needed to address the continuum of severity of alcohol dependence [16,17].

It could be argued that a chronic relapsing model might promote appropriate care for people with severe alcohol dependence (and other co-occurring health issues) in a specialized addictions setting. Dependence may not, however, be at the root of complicated and long-standing problems, and holistic orientations more appropriate for mainstream rather than specialist services may be called for when issues such as broken relationships, employment, housing, violence, physical health complications and co-occurring mental health issues come into play. It is also possible to envision chronic care packages which are attuned to the longer-term needs of the majority with dependence who often do not have complex co-occurring health issues and where extensivity rather than intensivity of intervention matters more [45,46]. Various researchers have been involved in the design and evaluation of continuing care protocols [47–49] which see ‘acute’ interventions at times of particular difficulty supplemented with brief efforts to support longer-term self-management. This clinical research effectiveness evidence base is, however, at an early stage of development, even though this may resemble how people actually do use treatment services, and health services research will also be needed to explore appropriate service provision models.

In a primary care setting, it is unknown whether the clinician may be more or less likely to ask about use of alcohol and intervene where indicated, if they think that alcohol dependence is a chronic relapsing disorder. While the therapeutically committed clinician who feels well supported in their role may be more likely to do so, others may be less likely to [50]. The majority of people who are identified with alcohol dependence in general practice will not be chronically relapsing nor display the wider needs of those in specialist treatment services. The label does not convey optimism about scope for positive change among either those with relatively moderate or severe dependence. There is a danger that it fosters low expectations which become self-fulfilling prophecies [51–53]. Brief treatments offered by generalists for those with dependence are in need of further development [54], as are online treatment interventions [55]. The concept that a chronic relapsing disorder model will lead to more and more appropriate treatment is an assumption that needs open consideration.

### For research

The chronic relapsing disorder model has its roots in the examination of people in specialized addictions treatment settings. There is profound selection bias here. Severity of alcohol dependence does drive people into treatment, but this is one influence among many. People in treatment are more likely to have other concurrent mental health and substance use concerns [56–58] as well as to have experienced other problems in their lives, particularly just prior to help-seeking [59–63]. Put simplistically, people choose to enter into treatment because they have problems (or are made to go because somebody else determines they have problems). Those in treatment often have a complex set of difficulties above and beyond their alcohol dependence and it may be these co-occurring problems that lead to the increased likelihood that they will relapse and show up in treatment repeatedly, rather than alcohol dependence *per se*[56,62,64–67]. Notwithstanding acknowledgement of extensive comorbidities and the dominant understanding of the multi-faceted nature of dependence, there is a tendency to see alcohol dependence as the root cause of many of the associated difficulties. It is rather odd that we base our understanding of alcohol dependence on those most severely afflicted by other problems. Perhaps we would do better to give greater emphasis in our attempts to understand dependence to the young adult populations where the majority of dependence is to be found, relative to middle-aged individuals who end up in treatment often beset by accumulated life problems [26,27,65]. These difficulties may have originated prior to the onset of dependence and make causal contributions to its development [68]. It is also worth considering whether alcohol dependence seen in young adults is something of a measurement artefact, an earlier manifestation of the same phenomenon seen at older ages or whether the onset of additional problems changes dependence in a fundamental way, or some combination of these possibilities [65,69,70].

There is a clear need to understand more effectively the development of alcohol dependence over the life-course in people who do not seek treatment. This is particularly true as it may well be that dependence is just as modifiable, or indeed more so, among those who do not attend treatment services. This can be seen in studies exploring the natural history of alcohol dependence [22,23,25,71]. Evidence-based interventions designed for use with treatment-seeking populations share important characteristics or may be the same as those effective for those not seeking help. For example, Motivational Enhancement Therapy, one of the most well-evaluated psychosocial treatments for alcohol dependence, is simply an elongated version of the Drinkers Check-Up, designed originally to support non-treatment change [72,73]. At least among those who change successfully, the social, behavioural and motivational mechanisms of change are probably very similar with and without the support of treatment services [71,74–77]. The problem is that we do not really understand what it is that differentiates people whose alcohol dependence will chronically relapse from those who will resolve it successfully by themselves or with a little-well designed help. The forthcoming revision of DSM-V combines the conceptually distinct domains of dependence and other types of problems in a new category of disorder [78]. We share concern that this will probably lead to a diminution of attention to problems other than dependence [79], and this will probably make the chronic relapsing disorder label even more inappropriate for those diagnosed as having disorders. It is unclear what the consequences of this change will be for the life-course study of drinking behaviour.

Finally, the adoption of the chronic relapsing disorder model appears to have implications for the allocation of resources for conducting research [80]. Regarding alcohol dependence as a chronic relapsing disorder may be one of the contributing factors to the ongoing biomedicalization of alcohol problems. It has been posited that the result of this biomedicalization is that the large majority of funding for research goes to biobehavioural research to the detriment of research exploring the psychological and sociological components driving alcohol dependence [80]. Given that dependence on alcohol is fundamentally a behaviour, albeit co-determined by the interaction of biological, psychological and social influences, this is a real problem.

## CONCLUDING REMARKS

Caution is needed when using the chronic relapsing disorder term, as the value of this perspective is diminished when one considers the problems probably attendant on its careless application. Restricting attention to those with ‘complicated alcohol dependence’, which may be a better way of thinking about and referring to this population, has profoundly adverse consequences for both science and clinical and public health interventions. Insufficient attention has been paid to the entire distribution of those with alcohol dependence. We believe that a chronic relapsing disorder model is not a useful conception for understanding the experience of the majority of people who have difficulties with alcohol dependence at some point in their life and that the influence of age and age-associated problems linked to dependence would benefit from further elaboration. Further, adopting a chronic relapsing disorder model may lead to an imbalance in considering the factors contributing to alcohol dependence, shifting focus away from psychological, social and environmental contributors towards an overemphasis on neurobiological perspectives. In addition, it is unclear whether a chronic relapsing disorder perspective serves some other useful purpose, such as leading to reduced stigma for those with alcohol dependence or to increasing the likelihood of accessing effective treatment. Until there is some evidence that adopting this model of alcohol dependence provides researchers, practitioners and policy makers with significantly more benefits than it does potential harms, we suggest that alcohol dependence itself should not be regarded as a chronic relapsing disorder.
